# Computational modeling of the cell-autonomous mammalian circadian oscillator

**DOI:** 10.1186/s12918-016-0379-8

**Published:** 2017-02-24

**Authors:** Olga A. Podkolodnaya, Natalya N. Tverdokhleb, Nikolay L. Podkolodnyy

**Affiliations:** 1 ICG SB RAS, Novosibirsk, Russia; 2 Novosibirsk State University, Novosibirsk, Russia; 3 ICMMG SB RAS, Novosibirsk, Russia

**Keywords:** Mathematical modeling, Mammalian circadian oscillator

## Abstract

This review summarizes various mathematical models of cell-autonomous mammalian circadian clock. We present the basics necessary for understanding of the cell-autonomous mammalian circadian oscillator, modern experimental data essential for its reconstruction and some special problems related to the validation of mathematical circadian oscillator models. This work compares existing mathematical models of circadian oscillator and the results of the computational studies of the oscillating systems. Finally, we discuss applications of the mathematical models of mammalian circadian oscillator for solving specific problems in circadian rhythm biology.

**Electronic supplementary material:**

The online version of this article (doi:10.1186/s12918-016-0379-8) contains supplementary material, which is available to authorized users.

## Background

Eukaryotic circadian clock is a timing system that forms rhythmic changes of all processes in the body, from molecular and genetic to physiological and behavioral, with a period close to 24 h. These oscillations allow organisms to adapt to the cyclic changes in their habitats [1].

Nearly a half of all mammalian genes change their expression levels in a circadian fashion [2, 3]. Therefore, any analysis of gene expression requires consideration for this kind of variations. However, the circadian mechanism studies are necessary for medicine. The disruption of this clock may cause a variety of pathologies, including cardiovascular and inflammatory diseases, cancer, depression, etc. [4–11]. The modern high-throughput experimental technologies supporting the genomics, transcriptomics, proteomics, metabolomics, and other ‘omics’ sciences, provide fundamentally new possibilities for the systems biology of the circadian clock. Mathematical modeling of the circadian clock provides a unified theoretical framework accounting for available experimental observations and allowing perform theoretical studies that are difficult to fulfill experimentally [12]. In particular, computational models and simulation experiments allow one:

- To clarify and to validate (or invalidate) particular working hypotheses;
- To analyze complex systems involving multiple variables which correlate to each other;
- To identify key interactions and parameters, and their qualitative or quantitative influence on the system’s behavior.
- To perform rapid, systematic exploration of the proposed mechanisms for circadian clock regulation in a wide range of conditions.
- To determine the conditions permitting a variety of oscillation-related phenomena, including conservation or disruption of oscillations, changing of oscillation period, phase shift, change in oscillation amplitude, etc.
- To generate testable hypotheses necessary for planning new experiments which could either validate the model or call for its modification, etc.

Adequacy and accuracy of the models depend on many factors, including the degree of understanding of given molecular system, the level of mathematical formalism, the structure of the model, the accuracy of estimation of the model parameters, etc.

In this review, we summarize and discuss the results of mathematical modeling of cell-autonomous mammalian circadian oscillator (CACO).

The first few sections provide basic understanding of the cell-autonomous mammalian circadian oscillator by summarizing experimental data essential for reconstruction and verification of CACO mathematical models and the highlights potential pitfalls in building and validation of mathematical models of circadian oscillator. Further, this review covers comparative description of existing CACO models as well as examples of applications of CACO to solve specific problems of practical importance.

### Mammalian circadian oscillator and its regulation

The mammalian circadian timing system is organized hierarchically by the circadian pacemaker localized in the suprachiasmatic nucleus (SCN) of the hypothalamus. This master pacemaker can synchronize the network of peripheral circadian oscillators in brain cells (outside SCN) and in peripheral tissues, however, underlying neural and humoral mechanisms remain obscure. Light is the main external stimulus that shifts the phase of the pacemaker. In fact, every cell in the organism contains an autonomous molecular-genetic circadian oscillator. Its structure can be described by a complex gene network and feedbacks mediated by transcription processes, post-translational modification of proteins, protein-protein interactions, chromatin modification, and others. It is generally accepted that the following seven gene groups - Clock (*Clock* gene and its homolog *Npas2*), Bmal (*Bmal1* and *Bmal2* genes), Per (*Per1, Per2* and *Per3* genes), Cry (*Cry1* and *Cry2* genes), CK1 (*CK1e* and *CK1d* genes) and Rev-erb (*Rev-erbα* and *Rev-erbβ* genes) and Ror (*Rorα, Rorβ* and *Rorγ* genes) encode minimal universal core of circadian oscillator [13–15].

**Fig. 1:**
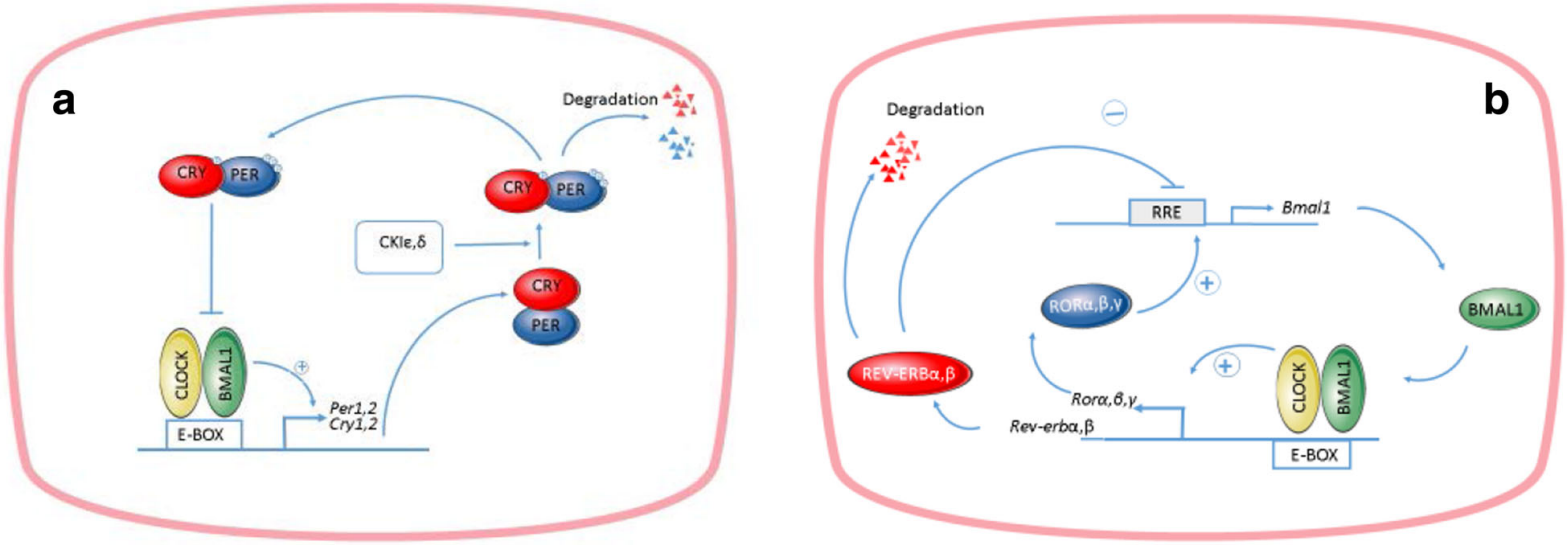
The minimal set of feedbacks providing functioning of the mammalian circadian oscillator: **a** the primary loop; **b** the stabilizing loop

The primary loop of negative feedback of circadian oscillator is formed upon activation of Per and Cry genes by transcription factor (TF) CLOCK:BMAL1 (Fig. 1a). The protein products of Per and Cry form PER: CRY heterodimers, which suppress activity of their own genes via protein-protein interactions with CLOCK:BMAL1 transcription factor [16–20]. Oscillations of TF CLOCK:BMAL1 activity occur with a period close to 24 h. An important role in establishing the oscillation period is played by post-translational modification of PER proteins by casein kinases CKIε and CKIδ [21, 22]. Another regulatory loop is induced by CLOCK:BMAL1 heterodimers by activating the transcription of genes Rev-erb and Ror (Fig. 1b), which, in turn, compete for RRE (Rev-Erbα/ROR response element) binding sites within Bmal1 and Clock gene promoters. While REV-ERBs repress the transcription process, RORs activate transcription [23–26]. Thus, RORs and REV-ERBs both positively and negatively regulate the circadian oscillation of Bmal1 and Clock, but to a lesser degree [26]. This feedback loop stabilizes rhythmic oscillations generated by the primary circuit [23, 27–29].

In addition, many other feedbacks are described in the literature, but these two loops are considered as the most basic.

The autoregulatory feedback loops described above can generate and maintain a stable circadian rhythm in a cell, while its phase can be shifted by external stimulus. This all makes circadian oscillator an important object for experimental and computer modeling aimed at discerning the principles of organization, behavior and characteristics of complex biological oscillators.

It is important to note that the idea of mammal circadian time control system being hierarchical is currently being revised. There is an opinion that it can be better described as a quasi-hierarchical. According to this line of thoughts, in addition to SCN, there are at least two additional pacemakers – methamphetamine sensitive circadian oscillator (MASCO) and food-entrainable oscillator (FEO) [30–32]. Moreover, two more non-canonical circadian oscillators were recently described in mice: wheel-inducible circadian oscillator (WICO) and palatable meal-inducible circadian oscillator (PICO) [33]. The locations and structures of newly described pacemakers are not known. However, it is suggested that these pacemakers are capable of compensating the function of SCN circadian oscillator and regulation of the rhythms of motion activity, endocrine activity and body temperature in absence of suprachiasmatic nuclei [33, 34].

### Circadian gene expression in mammalian tissue

The results of study of daily expression of genes from the circadian oscillator core are commonly used for the development of mathematical models of cell-autonomous circadian clock.

These data are necessary for finding the correlation of expression phases of the main components of oscillator and the genes regulated by it, for understanding the mechanisms for external stimuli entrainment of mammalian circadian clock and the role of each clock component in overall functionality of the molecular clockwork.

In addition, one can use them to explore the pathways through which the oscillator transmits and receives signals providing circadian synchronization of the processes regulated by it.

After the discovery of the role of mouse *Clock* gene in the mechanism of circadian rhythm production in SCN [35], the other genes of the mammalian circadian clock were found.

The development of modern high-throughput methods of gene expression analysis allowed to essentially expand the knowledge about circadian clock and circadian transcriptomes of different organs and tissues. Data on circadian dynamics of genes transcription in different tissues and cell lines can be found in the CircaDB database [36] or a public functional genomics data repository GEO (Gene Expression Omnibus) [37].

Identification of genes, whose expression follow circadian rhythm, and estimation of rhythm parameters can be performed by using methods based on various algorithms, such as JTK_CYCLE [38], Fisher’s G test, COSOPT [39], ARSER [40], CircWave [38–45] etc. COSOPT runs on Microsoft Windows, JTK requires R packages, and ARSER is implemented as a Python program calling some R functions. For COSOPT and JTK Cycle analyses, data is detrended by linear regression. The BioDare service can be used to estimate the period of circadian rhythms [46].

Nowadays the idea of rhythmic type of expression with a period close to 24 h for 3–15% of all mRNA in a particular tissue of mammals is generally accepted. Zhang et al. studied circadian dynamics of gene expression in 12 mouse organs and found that about 43% of genes coding proteins show the circadian rhythm of transcription at least in one organ [47]. Only limited set of genes oscillates in all examined organs simultaneously. However, even this set of genes varies from study to study, from 41 to 10 genes. One can explain it by differences in conditions of experiments, different sets of examined tissues, and peculiarities of data processing methods used by researchers [2, 39, 47, 48].

Generally, the data represented by most researchers indicate that mammalian circadian expression of majority of genes is tissue-specific. It reflects the physiological function of given tissue.

Most of the studies, which serve as a basis for current knowledge of circadian dynamics of gene expression, in fact, use the estimations of steady-state levels of mRNA assuming that rhythmic changes in mRNA levels reflect rhythmic changes of transcription of corresponding genes. The methods [49–52] allowing directly measuring the amount of nascent mRNA (Nascent-Seq and GRO-seq, NET-seq) help to re-examine this statement. It turned out that transcription as the main source of rhythmic expression formation at mRNA level characterizes only 20–30% of genes [49, 50]. Similar results were obtained by using these approaches in the study of Drosophila circadian gene expression [51]. For the remaining 80–70% of genes it is assumed that rhythms in gene expression are the result of regulatory events at multiple steps, such as mRNA splicing and degradation, nuclear export, methylation, translation, etc. [49, 52–55]. About 30% of transcripts of mouse liver and Drosophila head are under rhythmic post-transcriptional regulation, which is conducted in particular via regulation of mRNA degradation [56].

In addition, experiments of Menet et al. [49] showed a significant difference between the phases of CLOCK:BMAL1 DNA binding and the target gene transcription, including the transcription of key core clock genes, such as *Per1, Per2, Cry1, Rorγ*, etc. It is shown that CLOCK:BMAL1 binds with all target genes at the same phase of the cycle, though the peaks of transcription are heterogeneous and have no relations with the phase of CLOCK:BMAL1 DNA binding [49, 57]. Consequently the activity of other transcription factors supports the heterogeneous transcriptional output of CLOCK:BMAL1 target genes and this activity relies on the rhythmic regulation of chromatin accessibility of CLOCK:BMAL1 [49, 57].

With development of large-scale proteomic studies, it was found that expression patterns of many genes at the mRNA and protein levels often do not correlate. According to the different estimations, about 20–50% of the rhythmic proteins in the liver are accompanied by non-rhythmic mRNAs [58–60]. Among the circadian proteins themselves, only 18% remain circadian when their mRNAs are quantified [58–66].

These differences may also indicate the contribution of translation and post-translational modifications to the formation of circadian rhythm of expression of genes. First of all we should pay attention to degradation processes of protein and mRNA, as they may be not only the cause of differences in expression pattern of mRNA and proteins, but also provide the formation of circadian rhythms [56–63].

### Mutations in the circadian oscillator genes and the functionality of the circadian clock

One of the validation criteria for mathematical models of the circadian oscillator is the ability to predict phenotypes generated by oscillator nucleus genes mutations observed in vivo, in particular, the effect of mutations on SCN explants, their particular neurons, cells of peripheral tissues or the whole organism.

In Additional file 1, we present data on phenotypic effects of mutations of circadian oscillator genes in mouse.

Previously a specific characteristic of circadian clock was noted – it was clearly marked redundancy of homologous genes, which were supposed can functionally replace each other. However, the experiments on animal genetic models showed incomplete functional similarity of such genes (see Additional file 1).

Nevertheless, in the models *Per1*, *Per2*, *Per*3 genes are often represented as a one *Per* gene. In the same way, *Cry1* and *Cry2* are often presented as a one *Cry* gene (see Additional file 1). This approach definitely simplifies the modeling process; however, it may distort the overall picture.

In addition, we note that the manifestation of circadian clock mutation at the levels of organism, tissues and individual cells can differ and behavior does not necessarily reflect cell-autonomous clock phenotypes.

Thus, for example, Liu at al. [64] revealed that *Per1* ^−/−^ SCN explants exhibited a steady rhythm with period similar to WT in consistency with behavioral phenotype. *Cry1* ^−/−^ SCN explants also displayed a steady rhythm, but with a shorter period, consistent with behavioral phenotype as well. However, in dissociated individual SCN neurons the same mutations lead to the loss of circadian rhythm. These results were explained by presence of intercellular coupling in SCN neuron network, which has unique ability to compensate genetic defect of autonomous cellular clock and produce the rhythm close to normal in the explants or whole SCN, even in such conditions [64, 65]. While the rhythm generated by isolated neuron was determined by the condition of cell-autonomous oscillator and reflected its reaction to mutation. This is confirmed by similarity of changes caused by mutations in core circadian oscillator genes in isolated neurons SCN, isolated fibroblasts and peripheral tissues that lack of resistance to genetic disturbances (Additional file 1) [64]. Design and development of circadian oscillator model should take into account these differences.

### Building, identification, and validation of mathematical models of circadian oscillator

Building a model of a complex biochemical network is usually a time-consuming iterative process. At that, we need to take into account that data on individual reactions and data on functional states represent fundamentally different information, and the both types of information are equally important for the reconstruction of the model.

Based on the availability of data and desired analysis, modelling approaches in systems biology can be broadly categorized as bottom-up or forward hypothesis-driven modelling, top-down or inverse data-driven modelling approaches and middle-out which combines both strategies [66].

Bottom-up modelling methods often start from detailed mechanistic knowledge about the molecular structure of different molecular components of the circadian system, and then perform its assembly into larger units (e.g., a gene networks). A bottom-up model structure is defined a-priori by the modeler and model tends to be a physics-based model with many parameters. The modeler estimates each parameter of the model independently for small subsystems to combine them. A new processes can be included if the modeler thinks that they are important.

Top-down modelling approaches are trying to get the system characteristics beginning with observed data and comprising metabolic or gene network reconstruction via ‘omics’ data generated through RNA-Seq, DNA microarrays or other modern high-throughput genomic techniques using appropriate bioinformatics methodologies and statistical techniques.

These models are generally well suited to conceptual representation, and have as few parameters as possible in order to reproduce only the dominant response characteristics and easily identify them. The model structure is derived based only on available data.

The middle-out approach combines bottom-up and top-down modeling [66]. Therefore, one can take advantage of top-down modeling to determine the major drivers of circadian system and bottom-up modeling to understand detailed target mechanisms.

To combine the bottom-up and top-down approaches one can use process-based understanding obtained from bottom-up models to clarify the representation of processes in top-down models or use the results of computational experiments with the bottom-up model to determine the range of parameter values in a top-down model (or vice-versa) [66].

Parameter estimation from experimental data is an important step towards obtaining a “good” CACO model that can be used for the prediction and “what if” scenarios.

A necessary condition for parameter estimation is structural identifiability, also called a priori identifiability, theoretical identifiability, or qualitative identifiability, which suggests the possibility of evaluating the unique values for model unknown parameters from the available observables, presuming perfect experimental data (i.e. noise-free and continuous in time) [67–69].

Structural identifiability is a model property depending on the system dynamics, observable functions, external stimuli, and initial conditions. It does not depend on the amount or quality of the available data. In the absence of a unique correspondence between parameter values and the observed output of CACO model, a quantitative description of the biological process involving the unidentifiable parameter becomes impossible [68].

A number of analytical approaches to structural identifiability have been proposed, including Laplace transform (transfer function), Taylor series expansion, similarity transformations, differential algebra etc. (see reviews: [67–71]).

The basic concept in the model identifiability is a sensitivity that allows to determine which parameters are more important, i.e. have a greater effect in the model output, and to select the subset of identifiable parameters. Two aspects related to parameter sensitivities must be taken into account: their magnitude (i.e. a parameter cannot be identified if the models output is hardly sensitive to it) and correlation (i.e. two or more parameters cannot be estimated if their effects can be mutually compensated).

Classic sensitivity analysis allows determining the relative stability of model dynamics to certain parametric perturbations. For the limit-cycle oscillatory systems, including CACO model, as a rule, biologically relevant sensitivity analysis include [72, 73]:

- “shape” of the oscillation (in particular, oscillations amplitude range or size of oscillation area, etc.) response to a state perturbation (for example, the one-time addition of a clock protein), a permanent parameter change (for example, knockout of a clock gene or protein isoform) or temporary parameter perturbation (for example, applying a light pulse to photosensitive cells);
- phase or period response to perturbation;

The phase response of a system to state or parameter perturbations occurring at different phases is commonly called a phase response curve, and its amplitude equivalent may be called an amplitude response cure. By combining these sensitivity metrics with biological investigations, mathematical models may be used to guide experimentation, predict system behavior under new conditions, identify the roles of novel genes within the biological circuit, or uncover the mechanisms of drug action.

Assessing the structural identifiability of a model is only one aspect of the inverse problem that includes a priori or theoretical structural identifiability, a posteriori or practical identifiability and parameter estimation. Even in the most favorable case (when CACO model is structurally identifiable), it may not be possible to determine parameter values in practice [68] mainly from the insufficient number and quality of experimental observation for model fitting and / or due to model insensitivity to the parameter variability.

Practical identifiability or estimability is about quantifying the uncertainty in the estimated parameter values and calculating their confidence intervals, taking into account not only the model structure but also the information contained in the available data.

The models in systems biology are disproportionate in the relatively small amount of available data compared to the relatively large number of parameters in the rate laws [74]. Therefore, successful and accurate estimation of these parameter values is a critical part of CACO modelling, as the available experimental data tend to be determined with a large uncertainty or under environmental conditions different to the current experiment [75, 76]. On practice, this type of measurement is used for determination of biologically “reasonable” range, where the search for optimal estimations of the parameter values is conducted.

Before “omics” data were available, essentially all researchers used only ‘local’ kinetic information on biochemical or physiological processes to develop the models in the traditional reductionist manner [69].

When properly done this forward or bottom-up process results in a model that describes the same features as nature, if not quantitatively, but at least qualitatively. At the same time, this approach has some disadvantages, in particular, requiring a considerable amount of local kinetic data, which may be heterogeneous and may contain noise associated with different conditions of experiments, different organisms, different species, unaccounted factors and measurement errors. Therefore, the ‘integrated result’ infrequently is consistent with biological observations [69].

Essentially different method of parameter estimation from steady state data uses responses of a circadian system to small perturbations around the steady state.

To estimate the parameters one can minimize the cost function, which define the model accuracy by measuring parameter-dependent deviations between model behavior and experimental observations. Typical cost functions that work well in practice include the (weighted) least squares, maximum likelihood, and Bayesian estimators, in increasing order of the amount of information required to calculate them [70, 71].

Even if a model is identifiable, the fitting process itself may fail because either the optimum of cost function is local or even if a global optimum is found, there may be several suitable parameter sets.

Statistical validation of the model depends on the experimental data according to which the model was fitting. For a fixed set of experimental data, there is an optimal number of independent variables (parameters), which can be included in the model. It’s necessary estimate the adequacy of inclusion of each variable and parameter into the model.

In such cases, one can use independent cross-validation by separating data into training and test (validation) sets [77, 78]. After model parameters have been determined by fitting on the training set, the model validated by predictions against the test set. If predictions match, then the model is accepted. Otherwise - rejected. Essentially different strategy is to use wild-type data as training data and mutant data to test it [79].

Often the available data are not sufficient for proper model calibration. In this case, new data should be produced by new experiments to reduce the uncertainty in the estimated parameter values and obtain narrower confidence intervals for them. Experiments with the model allow the formation of new hypotheses, and suggest opportunities for design of new experiment, which will either validate the model or modify it. The reviews [80, 81] provides a broad overview of model-based experimental design methodologies for systems biology, including methods for various optimal parameters identification.

The estimation of the relative quality of models and model selection based on quantifying the degree of model complexity for a given data set can be based on Akaike information criterion or maximum likelihood method [82, 83].

The process of the model validation could include testing the model adequacy criteria of the circadian oscillator. The concordance of the characteristics of the system under modeling to the characteristics of the model under development is an important estimation criterion.

Biological rhythms are called circadian, if they meet a set of general criteria, which we expand and modify for model validation (Table 1) [79, 84–86]. Depending on the purpose of modeling an important role in validation of the computer CACO model can play various combinations of the above requirements and criteria. Clearly, any model with a limit cycle oscillations can show 24 oscillations through an appropriate re-scaling of time but an explicit representation of the time requirement eliminates many uncertainties at the coordination and interpretation of the various events and signals of the circadian oscillator system.

**Table 1:** Extended requirements in validation of computer CACO models

Criterion	Expected behavior of the circadian oscillator system
1. Circadian period close to 24 h	Without external influences, molecular oscillations of concentrations of RNA and proteins in CACO genes occur with a period close to 24 h. In diurnal animals, in general free-running period is slightly greater than 24 h, whereas, in nocturnal animals one is shorter than 24 h.
2. The phase concordance	a) Under autonomous (free running) conditions, the phases of oscillations of RNA and protein levels are concordant with each other and with experimental data. b) Circadian oscillations of the molecular concentrations occur with appropriate phase shifts to each other and to the light–dark cycle
3. The entrainment of circadian clock by incoming stimulus	There have to be a possibility to regulate the rhythms in response to external stimuli, a process called entrainment. Under influence of some periodic changes in environment (incoming stimulus) circadian rhythms can be delayed in frequency and phase. All circadian rhythms are synchronized by 24-h light–dark cycles. The closer period of the entrainment factor to the period of a free-running autonomous rhythm, the easier it entrained.
4. The reaction to the shift of daily rhythm	Rhythms can be adapted for concordance with local time. Under condition of phase shift of daily rhythm or constant lighting for daily light–dark cycle a new stable state of rhythm always comes after transition phenomena (the transition period), their duration is species-specific and can last several days. The closer the phase of the entrainment factor to the phase of initial rhythm, the easier and faster the delay occurs.
5. The reaction to a single light stimulus (phase synchronization)	(a) Single stimuli alter the phase of molecular oscillation. (b) The reaction of the circadian system to an external stimulus depends on the phase in which it is administered. This property describes the phase response curve. In diurnal species, exposure to light soon after wakening causes that the animal will tend to wake up earlier on the following day(s), whereas exposure before sleeping delays the rhythm, i.e. the animal will tend to wake up later on the following day(s). (c) The length and consistency of light exposure influences entrainment: - longer light exposures have more effect than shorter ones; - continuous exposure has a greater effect than intermittent exposure. (d) For wild-type mice, the phase shift after light stimulus can be equal to several hours (acceleration or delay). For mice with Clock^−/+^ mutation phase shift can reach 12 h.
6. Temperature compensation	The rhythms exhibit temperature compensation in mammalian. The period of circadian rhythms changes only slightly under different temperatures within the organism’s physiological range. If in enzymatic reaction Q_10_ value is usually changed from 2 to 3 (reaction rate increases 2–3 times as much under the increase of temperature on 10C^o^), then the circadian period does not change (Q_10_ ≈ 1) or even decreases (Q_10_ < 1) in mammalian. The temperature coefficient was calculated in the following equation: Q_10_ = (R_2_/R_1_)^10/(T2-T1)^, where R is rate and T is temperature.
7. Change of rhythm in gene mutations	Effect of mutations on the activity of circadian genes in vivo should be reproduced in the CACO model.

Finally, after model construction, one must determine the scope of a model, i.e., to what situations the model is applicable to or for which systems or situations the known data is a “typical” set of data.

In this section, we presented general challenges that modeling of circadian clock currently poses and a set of rules that help the modeling activity. The problems described in the modeling of circadian clock, in particular, the model validation, should get due attention; otherwise, resulting in a false prediction.

### Computational models of mammalian circadian oscillator

In 1965, when molecular mechanisms of circadian oscillator were not yet known, Goodwin [87] proposed a minimal phenomenological model of the generalized molecular oscillator describing the oscillatory negative feedback regulation of a protein, which inhibits its own transcription. The gene repression described in the form of a sigmoidal Hill curve, synthesis and degradation rates were linear.

Further, due to accumulation of new knowledge about the genes of circadian oscillator and features of their regulation new detailed computer models appeared. They contained up to several tens of biologically interpretable variables, including concentration of mRNA and proteins, which change depended on the rate of transcription, translation, degradation, modifications (phosphorylation, sumoylation, methylation, ubiquitination, acetylation and deacetylation etc.), formation and dissociation of complexes, transportation of cellular components, etc. [73, 87–93].

Typically, modern modelers use Michaelis-Menten equation, Hill function or protein sequestration to provide the necessary level of nonlinearity for circadian oscillations. The parameters in these models have a clear biological meaning: they denote the rates of synthesis, degradation or transport, binding affinities, etc. [11, 79, 86–102].

The Hill function may describe transcription processes, in particular, the protein complex repression or cooperative binding the repressor with gene promoter, and enzyme kinetics, in particular, the cooperative binding of multiple substrate or ligand molecules to an enzyme or a receptor. Hill coefficients in these processes are rarely higher than 3 or 4 [103]. However, Griffith demonstrated that Hill coefficients must be larger than 8 to obtain limit-cycle oscillations in Goodwin model [104]. In this regard, recently, modelers developed a new class of circadian clock models, which uses the protein sequestration-based transcriptional repression instead of the Hill-type repression [79, 94–102].

The need to integrate high-order nonlinearity or ultrasensitive response motifs in the model often arose in the simulation. Today, a number of ultrasensitive response motifs are known which can be generally divided into six categories: (i) homo-multimerization, (ii) positive cooperative binding, (iii) molecular titration, (iv) covalent modification cycle, (v) multistep signaling, and (vi) positive feedback [105]. These types of ultrasensitive response motifs may also be useful in modeling the circadian oscillator.

Next, we will focus on comparative analysis of the characteristics for some of the most important, from our point of view, mathematical models of the mammal circadian oscillator, including development goals, the initial assumptions that underline particular models, the used mathematical apparatus, model complexity and the results obtained by circadian oscillator modeling.

In 2003, Leloup and Golbeter develop the first computer CACO models of mammalian (the basic and the extended models) based on the interconnected negative and positive regulating feedbacks, including *Bmal1, Clock, Per* and *Cry* genes [88]. In these models *Per1*, *Per2* and *Per*3 genes were presented in the form of “unified” *Per* gene, as well as, *Cry1* and *Cry2* were presented as a *Cry* gene. The models described the process of transcription of these genes, formation and decay of CLOCK:BMAL1 and PER:CRY complexes, regulatory effects exerted on the gene expression by the BMAL1, CLOCK, PER, CRY, and REV-ERBa proteins, phosphorylation and transportation of proteins and protein complexes, degradation of mRNA and proteins, as well as light-induced Per expression [88].

The basic Leloup and Golbeter model contains 16 ODE, which present 16 variables and 55 parameters (see Additional file 2) [88]. According to experimental observations [1], the authors considered *Clock* expression constant and sufficient for providing a high concentration of CLOCK protein. TF CLOCK:BMAL1 activates transcription of *Per* and *Cry* genes. PER:CRY complex prevents this activation by binding to CLOCK:BMAL1 complex. Thus, expression of *Per* and *Cry* genes is indirectly inhibited by its own protein products. Despite the fact that proteins can be multiple phosphorylated [17], only one state of phosphorylation of PER, CRY, BMAL1 and PER:CRY complex are considered in the model. It is assumed that CLOCK:BMAL1 inhibits the transcription of *Bmal1* gene.

In the extended Leloup and Golbeter model the more detailed description of the influence of BMAL1 protein on expression of its own *Bmal1* gene is presented [88]. BMAL1 protein activates the expression of *Rev-erbα* gene, and REV-ERBα protein inhibits the expression of *Bmal1*. The extended model contains 19 ODE and 70 parameters. The authors manually choose model parameters with the values from the physiological range so that the oscillation period in the dark was close to 24 h. The parameters also satisfied other sets of limitations associated with the experimental observations, including the ability of synchronization for the oscillator by light.

As a result, both models showed the presence of autonomous circadian oscillations of *Per* and *Bmal1* in antiphase during the night. These models provided an opportunity to evaluate the ranges of parameter values for which the circadian oscillations were observed.

In addition, analysis of models allowed to suggest the multiple sources of periodic oscillations in the genetic regulatory network controlling circadian clock. Variants of conservation of oscillations or their disappearance at the gene knockouts were found.

Further analysis allowed to apply these models for the further study of different disorders of daily rhythm. In particular, it allowed to study the mechanism of rhythm disorder caused by Per2 gene mutations, the effect of PER2 phosphorylation reduction on period of the circadian oscillator, etc. The important result was a modeling of effectiveness for medications depending on time of their taking, influence of jetlag on circadian clock recovery [88, 106, 107].

Later, in 2003, Forger and Peskin developed another detailed regulation model of mammal circadian rhythms [85]. Biological basis of the model was obtained from the review article [1], which describes the classical idea of circadian rhythms regulation as a sequence of interaction of negative and positive feedbacks loops. In addition to the regulatory processes, the phosphorylation conducted with the help of proteins – casein kinase, which were included into the model. In particular, it is known that Casein kinase 1 epsilon (CK1ε) binds and phosphorylates PER proteins.

Ultimately, Forger and Peskin described in detail in their models:

1. the process of binding of CLOCK:BMAL1 transcription complex to a regulatory E-box element in *Per* promoter, assuming its independence from binding to the other regulatory elements;
2. the mechanism of transcription regulation by *Per2* and *Cry2* proteins, considering it as the same mechanism of transcription regulation by *Per1* and *Cry1* proteins;
3. the process of phosphorylation of PER1/PER2, assuming that: (a) the phosphorylation process can occur at many sites; (b) PER1 and PER2 non-phosphorylated proteins, located in the cytoplasm, are not able to bind to CRY and they degrade; (c) there are primary and secondary phosphorylations in the process of light-induction of *Per* transcription.

The total number of variables in the system of differential equations in Forger-Peskin model was 74 and the number of parameters was 36.

The fitting procedure based on optimization allowed to find a set of parameters for which model is in a very good agreement with the SCN data presented in [1] and liver data for the relative concentrations of the different clock proteins [17], due to the unavailability of SCN data. They included the null mutations of the PERs or CRYs genes in the model by setting the corresponding rates of transcription to zero. Removing PER2 allowed to abolish rhythmicity, but removing PER1, CRY1, or CRY2 severally didn’t, which is in agreement with experimental data (see Additional files 1 and 2).

In 2009, Forger and Peskin developed a stochastic model [108], which is a direct generalization of the deterministic mammalian circadian clock model [85]. The stochastic model of CACO was used for the following tasks: (i) comparing the behavior their deterministic and stochastic models; (ii) estimations on the accuracy of the clock within individual cells, and (iii) understanding what design principles contribute to robustness to molecular noise.

They found that [108]: (i) in certain cases, in particular, in the study of mutants, the stochastic and deterministic models exhibit qualitatively different behaviors; (ii) there are situations when a stochastic model oscillates, and the corresponding deterministic model does not; (iii) rapid interactions with promoters and multiple copies of genes reduce the variability of the period of the clock and increase the robustness.

By developing reduced mathematical CACO model [86] Becker-Weimann and coauthors have shown that negative regulatory feedbacks, which involve CLOCK:BMAL1, PER and CRY, are critically important for CACO functioning; and oscillations take place even if Ror/Bmal1/Rev-erbα regulatory feedback is replaced by activator with a constant expression.

Parameter variations that correspond to clock-gene knockouts reproduce experimental results, in particular, in mutant cells (*Bmal1* ^*−/−*^, *Rev-erb﻿α* ^*−/−*^ *, Per2* ^*Brdm1*^ */Cry2* ^*−/−*^ and the *Per2* ^*Brdm1*^ mutation) the oscillations do not occur (see Additional files 1 and 2).

The authors affirm that this is confirmed by the experimental data obtained in Rev-erbα^−/−^ mutations of mouse, they save CACO functionality despite the fact that regulatory feedback is disabled. However, it should be noted that later new experimental data was obtained. It revealed that for *Rev-erbα* there is a homologue – *Rev-erbβ* gene, which can conduct its functions in gene regulation of the circadian oscillator [109]; and apparently, this explains maintaining of CACO functionality in *Rev-erb*α^−/−^ cells. The model also accounts for the differential effect of the *Cry1* ^*−/−*^ and *Cry2* ^*−/−*^ mutations on the circadian period.

Due to the specific design taking into account only essential processes, model allows the use for various additional studies including [86, 110]:

- the entrainment of the circadian oscillator to light–dark cycles;
- extension of the model by incorporating the putative novel components or mechanisms;
- stochastic simulations for investigating the influence of molecular noise on circadian oscillations;
- analysis of the expression of different phases;
- the coupling of oscillators for the simulation of synchronization mechanisms;
- analysis of mechanisms of temperature compensation.

In 2009, Mirsky et al. developed another mathematical model of mammal circadian clock [90]. It contains 8 genes (*Per1*, *Per2*, *Cry1*, *Cry2*, *Clock*, *Bmal1*, *Rev-erb*, and *Rorϒ*) and thus describes more complete network and offers more opportunities for testing and validation of the model. In developing the model, the authors took into account the new exact phase correlations between molecular components identified in experimental studies at the intracellular level. These components reflect complex and often combinatorial regulation of circadian genes.

The model is implemented in MATLAB as system of ODEs, consisting of 21 equations with 132 parameters. Calculations were conducted on a computing cluster with the use of MATLAB Distributed Computing Toolbox. To describe the rate of transcriptions Michaelis-Menten kinetics are used, and mass action kinetics describe all the rest of rates (e.g., mRNA and protein degradation, formation and dissociation of complexes, etc.). Parameters adjustment is conducted by iterative evolutional algorithm with the focus on the intracellular phase interaction between the components of circadian oscillator. In addition to characteristics observed in circadian clock of autonomous cells at the molecular level, the model also describes various phenotypes of mutant cells (the knocked-out genes: *Per1*, *Per2*, *Cry1*, *Cry2*, *Bmal1*, *Rev-erb*, and *Ror*) (see Additional files 1 and 2).

In 2011, Relógio et al. developed one more model for the mammalian circadian clock. It allows studying two main contours of feedback: ROR/*Bmal*/REV-ERB and PER/CRY loops [91]. In the construction of the model, main attention was paid to a pacemaker in SCN, which is supposed to be responsible for synchronizing the whole circadian system and consequently can be responsible for general malfunctions and malfunctions of the peripheral clock that lead to rhythm disorders. The model was based on extensive research of literature and it took into account the experimental facts existing at the moment. In their model the authors combined the genes into following families: Per (*Per*1/2/3), Cry (*Cry*1/2), Ror (*Ror*α/β/γ), Rev-Erb (*Rev-erbα*/β), Bmal (*Bmal1*/*2).* The same principle was applied to the corresponding proteins and protein complexes.

Nonlinearity via Michaelis-Menten kinetics and the Hill function was introduced to describe transcription regulations. ODE system, gathered with the use of law of mass action and the linear kinetics of degradation, includes 19 ordinary differential equations with 71 parameters. Many parameters were found from the literature and others were estimated based on known amplitudes and phases.

To compare and joint use the amplitudes of different components found in the literature, the authors normalized the expression level of each component to its average value and the they were able to model expression profiles that oscillate near the baseline value of 1 for all variables that facilitated the comparison between them. Using the developed model, the authors were able to analyze the influence of transcription and degradation processes on CACO period and clarify the assumed role of dual-loop regulation of CACO.

In particular, it was studied the effect of increasing the rate of degradation of Per RNA, as well as of the rate of the clock proteins degradation on CACO period. The simulation showed that both increase and decrease in the oscillation period could be a consequence of these processes under certain conditions.

Another result of simulation is the prediction of damping of oscillations at high expression of components of the stabilizing loop of the oscillator (ROR / Bmal / REV-ERB). These predictions were confirmed by in vivo experiments.

Jean-Paul Cometa and coauthors [111] reduced the model of Leloup and Goldbeter [73] without loss of critical information. The reduced model included only 8 equations, and allows more carefully investigate basic mechanisms of governing the CACO.

In 2014, Jolley et al. developed a minimal mathematical model of CACO where regulation of transcription occurs through the interaction of the three regulatory elements: E-box, D-box and RRE. The E/E’-box responsible for gene transcription in the morning; the D-box, governs of the daytime transcription; and the RRE, in the evening.

In particular, the model describes the transcriptional regulation of *Cry1* gene through the D-box and RRE regulatory elements. Also this model to permits prediction of phase response curves based on ensemble regulatory elements.

The model has been validated using differential evolution for optimization. This model exclude many of the redundancy of the real CACO and exhibits less resistance to gene knockouts than the actual system [112].

Korenčič A., et al. [92] developed a minimal mathematical model, which allowed describing CACO in different tissues (liver, heart and adrenal gland) and under various lighting regime (DD and LD). The model included three feedback loops of CACO (with E-boxes, RRE-elements, and D-boxes). As a first simplification of the model, all insufficiently characterized interim steps (post-translational modifications, complex formation, and nuclear localization) were combined into one step with delay for several hours. Thus, the amount of kinetic parameters was significantly reduced. Then redundant regulators were combined based on their own gene expression measurement data. In particular, it is assumed in the model that the transcription of 5 genes included in the CACO model (*Per2*, *Dbp*, *Bmal1*, *Cry1*, *Rev-erb*α) is determined by basic circadian regulatory elements of the promoter: E-boxes, D-boxes and RRE. The final model is described by a system of differential equations with delays, which were estimated by difference between phases with the maximum level of gene expression and phases of maximum rate of protein production. Their values were determined experimentally. Comparative analysis of ChIP-seq experimental data for BMAIL1, REV-ERBα and REV-ERBβ showed that constructed CACO model allows us to describe distribution of maximum expression phases of genes under BMAL1 and REV-ERBα regulation.

The main factor that determines the phases of gene expression is E-box-regulated transcription. Although the other factors (D-box regulators, HSF, SRF, CERB, periodic degradation of proteins, their polyadenylation and regulation of ribosomal biogenesis) also influence the gene expression phases.

The model also shows that the multiplicative effect of E-boxes, D-boxes and RRE leads to appearance of harmonics that are different from 24 h, in particular observed in experiment 12-h peaks of gene expression (about 1% of all genes) in the liver. Computational experiments also showed differential regulation of cytochrome p450 gene.

Comparison of maximum gene expression phases in different tissues showed that the distribution of phases of CACO genes peaks in the heart differs from the same in other tissues, and this can be connected with the rhythm of tissue-specific transcription factors regulating CACO genes. In particular, in the heart they are the following: *Atf6*, *Gata6*, *Gtf2a1*, *Hif1a*, *Mef2a*, *Nfyb*, *Rbpj*, *Smad7*, *Tcf4*, *Tead4* [92].

In 2012, Kim and Forger [79] modified and significantly expanded model of the deterministic mammalian circadian clock developed by Forger and Peskin [85]. The new model included key genes, mRNA, and proteins, which are currently considered as central for the daily timekeeping of mammals (PER1, PER2, CRY1, CRY2, BMAL1/2, NPAS2, CLOCK, CKIε/δ, GSK3β, Rev-erbα/β). Despite the fact that only 10 monomers are involved, they can produce a multitude of complexes depending on their binding state, phosphorylation and subcellular location. As a result, the expanded model is presented in the form of an ODE system, which includes 75 parameters and 181 variables, including 159 variables for protein complexes; 12 variables for mRNA; 8 variables - identifiers of promoter activity and 2 variables for describing the effect of light and GSK3β activity.

The model parameters were estimated on the base of functional optimization by simulated annealing (global stochastic optimization method) that minimizes the difference between model simulations and experimental data. In particular, the authors took into account the experimentally determined rate constants, fit the experimentally observed dynamics of change in the concentrations of mRNA and proteins and fit the relative abundance of proteins.

The authors noted that their model is significantly better at predicting different phenotypes of gene mutations that take central place in CACO in comparison with existing models. The model also predicts mutant phenotypes *Rev-erbα* ^*−/−*^ *, Cry1* ^*−/−*^ *, Cry2* ^*−/−*^ *, NPAS2* ^*−/−*^ *, Bmal1* ^*−/−*^ *, Clock* ^*Δ19/Δ19*^ of isolated SCN neurons, which differ from SCN slice (see Additional files 1 and 2. Supplement).

Exploring the model behavior, the authors identify the following key mechanisms providing 24-h rhythms in circadian clock of higher organisms:

1. The correct stoichiometric ratio of activators and repressors. The stoichiometry is the average ratio of repressors concentration (all forms of PER and CRY in nucleus) to activators (all forms of BMAL:CLOCK/NPAS2 in nucleus) during the period.
2. A strong binding between repressors and activators.
3. A presence of supersensitive reactions close to 1–1 stoichiometry.
4. The half-life period of activators longer than that of repressors.

The authors also note that additional negative feedback loop is neither independent nor an auxiliary generator, but it plays its role in stoichiometry regulation and thus increases the robustness of rhythm and period.

Yan et al. use modeling of the CACO to study the relationship of the main negative feedback loop of oscillator with its additional loops and identify the possible mechanism of coordination of the interrelated loops of CACO to regulate the period and maintain its robustness [93].

They have used the comprehensive model with 6 genes (*Bmal1, Per1, Cry1, Per2, Cry2*, and *Rev-erba*) to confirm the above ratio hypothesis. In order to focus on the transcriptional regulations, they assumed that the post-translational time delay of each gene is fixed as an explicit time delay.

The model includes the following processes:

1. The regulations of PLBS activity and RORE activity.
2. Transcriptions of *Per1, Per2, Cry1, Cry2, Bmal1, Rev-erb*α genes.
3. Translations of *Per1* mRNA, *Per2* mRNA, *Cry1* mRNA, *Cry2* mRNA, *Bmal1* mRNA, *Rev-erb*α mRNA.
4. Post-translational regulations.

Eventually, the mammalian circadian model includes a set of delay differential equations and algebraic equations. During the exploration of the model parameters, they found that the post-translational time delays are the main factors, which significantly change the period of the oscillation. Therefore, the time delay of each gene is well estimated from the experimental data. Since other parameters do not significantly affect the period, authors chose these parameters in a proper range. The numerical simulation is performed in MATLAB (Mathworks) with a solver for delay differential equations (DDE23).

Yang and coauthors found an interesting regularity: the intensity ratio of the CACO primary loop to the stabilizing loop is inversely to the length of the period. This pattern is retained under conditions of a fixed post-translational feedback. The results obtained with this computational model have found experimental confirmation [93].

In 2016, Woller and coauthors have constructed a mathematical model of the mammalian liver circadian clock which incorporates the metabolic sensors SIRT1 and AMPK [113]. This model integrates feeding and fasting cycles with the circadian clock. It consists of 16 ordinary differential equations describing the time series of the mRNA and protein concentrations for the clock genes *Bmal1, Per, Cry, Rev-Erb, Ror*, the metabolic gene *Nampt*, the mRNA concentration for the clock output gene *Dbp,* and the NAD+ level. Model contains 96 kinetic constants, most of which are yet unknown and should be derived from experimental data. To describe the gene transcription, authors employs Hill function. The model accurately reproduces high-fat-diet-induced loss of NAD+ oscillations and predicts that this effect may be pharmacologically rescued by timed administration of REV-ERB agonist. The comparison of period length between the experimental data and the simulation result was carried out in follow genotype: WT, *Per1* ^*−/−*^, *Per2* ^*−/−*^ *, Cry1* ^*−/−*^ *, Cry2* ^*−/−*^ *, Rev-erbα* ^*−/−*^ *, Fbxl3* ^*−/−*^ *, Rev-erbα* ^*−/−*^ */Fbxl3* ^*−/−*^ *, Bmal1* ^*−/−*^ (see Additional files 1 and 2)*.*

Information about models of the circadian oscillator described in this section, including the year of publication, mathematical apparatus, the number of variables, the number of parameters, genes in the models, model assumptions, the experimental data, and the main results are shown for comparison in Additional file 2.

### Application of circadian oscillator models to biomedical problems

Mathematical models of the circadian clock were useful for designing experiments aimed at understanding of novel clock gene, the mechanisms of the pharmacological control of circadian rhythms, temperature compensation, ability to synchronize CACO and gene networks with different functionality, etc.

Various modifications of the detailed mathematical model of the mammalian CACO developed by Kim and Forger [79] were successfully used to address these challenges.

In particular, Goriki and colleagues [98] used an extended version of this model to confirm that the gene *CHRONO* can indeed be considered as an important component of the CACO. Studied *in silico*, this model confirmed that the behavioral *CHRONO* KO phenotype is an outcome of the observed biochemical features of Chrono. The model also predicts that CHRONO can determine the residual rhythmicity in *Cry1* ^*−/−*^ */Cry2* ^*−/−*^ cells [98].

Kim and coauthors [94] proposed a mathematical model that accurately predicts effects of joint action of two independent signals (pharmacological agent and light) on a circadian timekeeping. They extended the computer model of mammalian CACO [79] by including multi-compartment pharmacokinetic / pharmacodynamic model.

This modification correctly described the disposition of CK1δ/ε inhibitor PF-670462 and its interaction with CK1δ/ε.

Novel model allowed to predict that a stable phase delay can be produced by chronic CK1δ / ε inhibition during the earlier hours of the LD cycle.

However, in case of chronic day-time dosing, or upon longer light intervals, model did not yield an entrained rhythm [94]. The experimentally validated results of modeling indicate that exact pharmacological manipulation of phase circadian clock requires careful selection of the timing, dosing and environmental signals [94].

Zhou, Kim, and coauthors [100] presented the model, which is another extension of the model of Kim and Forger [79]. Authors proposed a phospho-switch model, where two competing phosphorylation sites of the protein PER2 determine whether it has a fast or slow degradation rate. To include the phosphor-switch in the extended model they added following processes to the original model: (1) degradation of unphosphorylated PER2 or phosphorylated PER2 at FASP sites; (2) degradation of phosphorylated PER1/2 at CRY binding site by CK1; (3) degradation of phosphorylated PER2 at β-TrCP binding site by CK1; (4) phosphorylation of PER2 by priming kinase; (5) phosphorylation of PER2 by CK1; (6) phosphorylation of PER2 by GSK3 [100].

The model reproduces experimentally revealed the kinetics of degradation of PER2 protein and explains the phenotypes of mutations CK1ε *tau* and FASP, which affect the phosphorylation of PER2. The model also predicts a critical role of phosphoswitch in temperature compensation [100].

Adaptation and modification of the detailed mathematical model of the mammalian circadian clock developed by Kim and Forger [79] allowed D’Alessandro and coauthors to justify the design of artificial circadian oscillator and to predict its behavior, which is able to generate tunable, robust circadian rhythms and can function in vivo and control natural circadian physiology [96]. This artificial oscillator is tunable, so one can predictably modulate the circadian period and phase.

D’Alessandro and coauthors predicted that the only component of the circadian clock, which can be used in a tunable synthetic oscillator, is PER. The design principles used in this work can be helpful in the development any synthetic systems with the properties of switches and oscillators which capable to control behavior in vivo [96].

DeWoskin, Myung and coauthors (2015) presented the results of studying the mechanisms of coupling between neurons within the SCN and modulation synchrony its neurons [102, 114]. They combined previously published models: 1) the molecular clock model [79]; 2) the VIP coupling model [104]; 3) the electrophysiology model [115]; 4) the GABA coupling model [104] to create a full detailed SCN model.

This integrated model of the SCN network can be a useful tool for studying the interactions between the molecular and electrical activity of single neurons in the SCN, synchronization processes and phase relations identified within the network [102].

The model was used to study the role of the neurotransmitter GABA in synchronizing circadian rhythms among neurons in SCN, and to search for the mechanisms of encoding the length of the day within the SCN neuronal network [102, 114].

Many of studies cited above in this section use a detailed model of the circadian oscillator of Kim and Forger [79] as the base for design of new models [94, 98, 102, 114]. One of the benefits of this model is a detailed description of the components and regulatory processes of the oscillator. This allows one to expand and to modify the model for simulation of various processes in accordance with the task. The results obtained using such models were related to various aspects of circadian clock functioning and helped to design experiments and to generate hypotheses later tested in vivo. At the same time, such models are very complex and often require large computing resources [102, 114]. Therefore, to solve specific problems, especially in the study of interaction of CO with other systems, in the field of medicine and chronopharmacology such detailed circadian oscillator models are not always required.

The results obtained using compact models also have a biological interpretation and answer to the questions posed in the studies. For example, compact models are used in chronopharmacology and chronotherapy.

In particular, Hirota and coauthors used such type of model in searching for therapy of chronopathology and in particular to investigate the potential mechanisms of action of small molecules KL001 (specifically interacts with cryptochrome (CRY)) and Longdaysin (CKI inhibitor) [116–118]. They showed that KL001 is a useful tool to study the regulation of CRY-dependent processes and may to aid in development of chronopharmacology. In addition, these models allowed establishing differences between FBXL3- and CKI-mediated clock regulations. Based on the same principles, models for identification of circadian determinants of cancer chronotherapy were designed [119–122].

One of the very important directions of use of mathematical models is the synchronization of CACO and gene networks with different functionality.

Tareen and Ahmad developed the computer model for simulating the effects of different feeding regimens, showed how the circadian system entrains to the feeding regimens, and simulated the changes in abundance of each protein involved in this circadian system [123].

The results of computer modeling for relations of cell cycle and circadian oscillator at molecular level can be found in several studies [124–127]. By using computational models for the circadian clock and cell cycle, the authors investigated the conditions in which the mammalian circadian clock can entrain the cell cycle. The formation of the complex oscillatory dynamics of the cell cycle (complex periodic oscillations, or chaotic oscillations) may be a consequence of the interaction of these two gene networks. It was revealed that at different stages of the cell cycle circadian clock regulate various cyclin-dependent kinases. Hence, circadian clocks are an important mechanism for temporal organization of the cell cycle [124–127].

Bratsun and coauthors [128] proposed a minimal multiscale chemo-mechanical model of cancer tumor growth induced by circadian rhythm disruption in epithelial tissue.

The model includes a division of cells and intercalation, as well as mechanical interactions and a chemical signal exchange between neighboring cells that allows to find the respective parameters for transformation into the cancerous state.

All of studies cited in the section were carried out using an integrated approach combining mathematical modeling and experiments.

## Conclusion and outlook

During the last decades, extensive researches lead to understanding the circadian timing system in all its facets, including a detailed study of the core circadian clock genes, a large-scale search for novel candidate circadian genes and circadian controlled transcriptional regulators and its direct targets, protein-protein interactions, molecular structure of circadian clock proteins etc. [129]. This knowledge could be used in medicine, chronotherapy, preventing disorders of biological rhythms, jet-lag and shift work, etc. [122, 130–132].

Mathematical modeling in conjunction with molecular-biological studies could be a powerful approach providing generation of hypotheses and predictions for future experimental tests.

Now we know that the dynamics of the expression of many genes at the level of mRNA and protein often do not correlate. Thus, it is necessary to clarify the mechanisms of forming their oscillation in CACO model. In particular, we should pay attention to degradation processes of protein and mRNA, translation and post-translational modifications, as they may be not only the cause of differences in expression pattern of mRNA and proteins, but also provide the formation of circadian rhythms of their expression.

A large number of mathematical models of mammalian circadian oscillator have been developed, but there are no suitable models for many real-life situations.

The developed models made a significant contribution to the understanding of the structure of circadian clock self-sustaining mechanism, the functional significance of its individual components, mechanisms of interaction of the circadian oscillator with the other functional systems of the organism. Novel types of experimental data, and also new application fields require the modification of already developed mathematical models or the creation of new ones.

The experimental facts about the circadian oscillator as well as the phenotypic effects of circadian gene mutations, the methods for model validation, CACO models review and their applications presented in this review may be useful for development of new models and its applications.

We should note that the modeling of the mammalian circadian clock depending on the specific tasks and subsequent analysis could be performed at different levels, including:

1. Modeling of cell-autonomous circadian oscillator (CACO).
2. Modeling of interaction and synchronization for cell-autonomous circadian oscillators in particular tissue;
3. Modeling of organism circadian clock, including the synchronization process of peripheral oscillators by central circadian oscillator in particular tissues;
4. Modeling of relationships between circadian clock and body functioning (sleep disorders, circadian rhythm of various biochemical processes, rhythms of sleep / wake, maximum working efficiency, etc.) depending on external action, etc.

Systematic study of influence of circadian rhythms on basic functions of the organism requires the use of higher-order computer models. Nevertheless, the models of circadian oscillator in a single cell are still useful for studying the systemic effects. Reasonable biological interpretation of the modeled changes of mRNA or protein concentrations is of paramount importance for integration of the modeled data into the organism-level chronobiology view.

The awareness to the disruption of the circadian rhythms as a contributor to many chronic diseases, in particular, neuropsychiatric conditions, cancer, type 2 diabetes and obesity calls requires continuations of chronobiology research efforts [122, 130–132]. Good example of this kind would be the biological data-driven mathematical model developed by Korenčič and coauthors [92]; this model provides insights into the tissue-specific regulation of circadian rhythms. Another important avenue for the chronobiology is the analysis of the effectiveness of medication depending on the timing of administration.

## Additional files

Additional file 1: Table S1. Phenotypic effects of circadian mutations. (DOCX 22 kb)

Additional file 2: Table S2. The comparative characteristics of mathematical models of the mammals CACO. (DOCX 22 kb)

## Abbreviations

CACO: Cell-autonomous mammalian circadian oscillator
CK1ε: Casein kinase 1 epsilon
FEO: Food-entrainable oscillator
GEO: Gene Expression Omnibus
MASCO: Methamphetamine sensitive circadian oscillator
PICO: Palatable meal-inducible circadian oscillator
RRE: Rev-Erbα/ROR response element
SCN: Suprachiasmatic nucleus
TF: Transcription factor
WICO: Wheel-inducible circadian oscillator
