# Supplementary material for: Computational modeling of the cell-autonomous mammalian circadian oscillator
Source: BMC Syst Biol. 2017 Feb 24;11(Suppl 1):27–42. doi: 10.1186/s12918-016-0379-8 (PMC5333193; doi:10.1186/s12918-016-0379-8)
Supplement: Supplementary file 1 — Phenotypic effects of circadian mutations. (DOCX 22 kb) [file 12918_2016_379_MOESM1_ESM.docx]

Additional file 1. Phenotypic effects of circadian mutations

| **Gene/model** | **Behavioral phenotype** | **Tissue / cellular phenotype** | **References** |
| --- | --- | --- | --- |
| *Clock^Δ19/Δ19^* | Longer pd (28 h) / arrhythmic | - | Vitaterna et al., 1994  Shearman LP et al. 2000 |
| *Clock*^-/-^ | Shorter pd (23.5 h) | Similar to WT: SCN  Arrhythmic: lung, liver | DeBruyne et al., 2007а  DeBruyne et al., 2007в |
| *Npas2*^-/-^ | Shorter pd (22 h) | Similar to WT: lung, liver, SCN | Dudley et al., 2003  DeBruyne et al., 2007в |
| P*er2^Brdm1^* | Shorter pd (22.5 ч.) / arrhythmic | Arrhythmic: SCN | Shearman et al., 2000b  Zheng et al., 1999  Zheng et al., 2001 |
| *Per2^ldc^* | Shorter pd (22.95 ч.) / arrhythmic | Similar to WT (reduced level of expression): SCN  Unstable rhythms of low amplitude or arrhythmic: fibroblasts | Bae et al., 2001;  Liu et al., 2007 |
| Per2^−/−^ | Shorter period (22 h) Arrhythmic in constant darkness | - | Bae et al., 2006 |
| *Per1^ldc^* | Shorter period (23.5 ч.) / arrhythmic  Similar to WT (Liu) | Similar to WT: SCN;  Arrhythmic: fibroblasts, lung, dissociated fibroblast cultures and dissociated individual SCN neurons | Bae et al., 2001;  Liu et al., 2007 |
| *Per1*^-/-^ | Shorter period | Similar to WT*,* phase delay*:* kidney, heart, skeletal muscle | Cermakian et al., 2001; |
| *Per1^Brdm1^* | Shorter period (22.6 h) with reduced precision and stability. | Similar to WT: SCN, liver, kidney | Zheng et al., 2001 |
| *Per1^Brdm1^* / *Per2 ^Brdm1^* | Arrhythmic | -- | Zheng et al., 2001 |
| Per3^-/-^ | Similar to WT / Short period (23.5h) | Short period: fibroblasts, SCN, lungs | Shearman et al., 2000b  Liu et al., 2007 |
| *Cry2*^-/-^ | Longer period (25 h),  delayed free-running periodicity | Longer period: SCN, lungs, liver, cornea, dissociated fibroblast cultures and dissociated individual SCN neurons | Van der Horst et al., 1999;  Vitaterna et al., 1999  Liu et al., 2007 |
| *Cry1*^-/-^ | Shorter period (23 h)/ Arrhythmic  Accelerated free-running periodicity | Shorter period: SCN;  Arrhythmic: lungs, liver, cornea, dissociated fibroblast cultures and dissociated individual SCN neurons | Van der Horst et al., 1999; Vitaterna et al., 1999  Liu et al., 2007 |
| *Per2^Brdm1^* / *Cry1*^-/-^ | Arrhythmic | - | Oster et al., 2002 |
| *Per2^Brdm1^* / *Cry2*^-/-^ | Similar to WT | - | Oster et al., 2002 |
| *Cry1*^-/-^ / *Cry2*^-/-^ | Arrhythmic in constant darkness | Arrhythmic: SCN, lungs, liver, cornea  Abolished acute light induction of mPer1 mRNA in the SCN;  Acute light induction of mPer2 in the SCN was intact | van der Horstet al., 1999; Vitaterna et al., 1999  Shearman et al. 2000  Liu et al., 2007 |
| *Bmal1*^-/-^ | Arrhythmic in constant darkness | Arrhythmic: fibroblast, SCN neurons;  Persistent, yet highly variable, rhythmicity: SCN explants | Bunger et al., 2000  Ko et al.,2010 |
| *CKI*ε^tau/tau^ | Shortened period (20 ч.) | Shorter period: SCN, lungs, kidneys, pituitary | Meng et al., 2008 |
| *CKI*ε^-/-^ | Lengthened period | Similar to WT: fibroblasts, SCN, liver | Etchegaray et al., 2009  Etchegaray et al., 2010  Meng et al., 2008 |
| *CKI*δ *^Δ2/Δ2^* | Viable | Lengthened period: fibroblast cultures, SCN explants, liver | Etchegaray et al., 2009  Etchegaray et al., 2010  Lee et al., 2011 |
| *CKI*δ *^-^*^/+^ | Shortened period (23.5 ч.) | - | Xu et al., 2005 |
| *CKI*ε^-/-^ / *CKI*δ *^Δ2/Δ2^* | Viable | Arrhythmia: fibroblasts | Etchegaray et al., 2009  Lee et al., 2011 |
| Rev-erbα-/- | Shortened period in constant darkness ( (23.5 ч.), disrupted photic entrainment | Similar to WT mRNA rhythm *Per2*, *Cry2, Clock;*  Arrhythm*ic Bmal1, Clock, Cry1, mRNA:* liver  Arrhythm*ic Bmal1:* SCN | Preitner et al.,2002 |
| *Rorα^staggere^* | Shortened period (23.5 ч.), disrupted photic entrainment | Similar to WT: fibroblasts | Sato et al.,2004 |
| *Ror*β^-/-^ | Slightly longer circadian period, (24.5 h) | - | André et al., 1998 |

**References**

1. Vitaterna MH, King DP, Chang AM, Kornhauser JM, Lowrey PL, McDonald JD, Dove WF, Pinto LH, Turek FW, Takahashi JS. Mutagenesis and mapping of a mouse gene, Clock, essential for circadian behavior Science. 1994;264(5159):719-25.
2. Shearman LP, Sriram S, Weaver DR, Maywood ES, Chaves I, Zheng B, Kume K, Lee CC, van der Horst GT, Hastings MH, Reppert SM. Interacting molecular loops in the mammalian circadian clock. Science. 2000;288(5468):1013-9.
3. DeBruyne JP, Weaver DR, Reppert SM CLOCK and NPAS2 have overlapping roles in the suprachiasmatic circadian clock. Nat Neurosci. 2007а;10, 543–5.
4. DeBruyne JP, Weaver DR, Reppert SM. Peripheral circadian oscillators require CLOCK. Curr Biol. 2007б: 17: R538–9
5. Dudley CA, Erbel-Sieler C, Estill SJ, Reick M, Franken P, Pitts S, McKnight SL. Altered patterns of sleep and behavioral adaptability in NPAS2-deficient mice. Science. 2003; 301: 379–83.
6. Shearman LP, Jin X, Lee C, Reppert SM, Weaver DR. Targeted disruption of the mPer3 gene: subtle effects on circadian clock function. Mol. Cell. Biol. 2000b:20, 6269–75.
7. Zheng B, Larkin DW, Albrecht U, Sun ZS, Sage M, Eichele G, Lee CC, Bradley A. The mPer2 gene encodes a functional component of the mammalian circadian clock. Nature. 1999 Jul 8;400(6740):169-73.
8. Zheng B, Albrecht U, Kaasik K, Sage M, Lu W, Vaishnav S, Li Q, Sun ZS, Eichele G, Bradley A, Lee CC. Nonredundant roles of the mPer1 and mPer2 genes in the mammalian circadian clock. Cell. 2001;105(5):683-94
9. Liu AC, Welsh DK, Ko CH, Tran HG, Zhang EE, Priest AA, Buhr ED, Singer O, Meeker K, Verma IM, Doyle FJ 3rd, Takahashi JS, Kay SA. Intercellular coupling confers robustness against mutations in the SCN circadian clock network. Cell. 2007;129(3):605-16.
10. Cermakian N, Monaco L, Pando MP, Dierich A, Sassone-Corsi P. Altered behavioral rhythms and clock gene expression in mice with a targeted mutation in the Period1 gene.EMBO J. 2001;20(15):3967-74.
11. van der Horst GT1, Muijtjens M, Kobayashi K, Takano R, Kanno S, Takao M, de Wit J, Verkerk A, Eker AP, van Leenen D, Buijs R, Bootsma D, Hoeijmakers JH, Yasui A. Mammalian Cry1 and Cry2 are essential for maintenance of circadian rhythms. Nature. 1999;398(6728):627-30.
12. Vitaterna, M. H., C. P. Selby, T. Todo, H. Niwa, C. Thompson, E. M. Fruechte, K. Hitomi, R. J. Thresher, T. Ishikawa, J. Miyazaki, J. S. Takahashi, and A. Sancar Differential regulation of mammalian Period genes and circadian rhythmicity by cryptochromes 1 and 2. Proc. Natl. Acad. Sci. USA. 1999; 96:12114–12119.
13. Oster H, Yasui A, van der Horst GT, Albrecht U. Disruption of mCry2 restores circadian rhythmicity in mPer2 mutant mice. Genes Dev. 2002;16(20):2633-8
14. Bunger MK, Wilsbacher LD, Moran SM, Clendenin C, Radcliffe LA, Hogenesch JB, Simon MC, Takahashi JS, Bradfield CA. Mop3 is an essential component of the master circadian pacemaker in mammals. Cell. 2000;103(7):1009-17.
15. Ko CH, Yamada YR, Welsh DK, Buhr ED, Liu AC, Zhang EE, Ralph MR, Kay SA, Forger DB, Takahashi JS. Emergence of noise-induced oscillations in the central circadian pacemaker. PLoS Biol. 2010;8(10):e1000513.
16. Meng QJ1, Logunova L, Maywood ES, Gallego M, Lebiecki J, Brown TM, Sládek M, Semikhodskii AS, Glossop NR, Piggins HD, Chesham JE, Bechtold DA, Yoo SH, Takahashi JS, Virshup DM, Boot-Handford RP, Hastings MH, Loudon AS. Setting clock speed in mammals: the CK1 epsilon tau mutation in mice accelerates circadian pacemakers by selectively destabilizing PERIOD proteins. Neuron. 2008;58(1):78-88.
17. Etchegaray JP, Machida KK, Noton E, Constance CM, Dallmann R, Di Napoli MN, DeBruyne JP, Lambert CM, Yu EA, Reppert SM, Weaver DR. Casein kinase 1 delta regulates the pace of the mammalian circadian clock. Mol Cell Biol. 2009;29(14):3853-66.
18. Etchegaray JP, Yu EA, Indic P, Dallmann R, Weaver DR. Casein kinase 1 delta (CK1delta) regulates period length of the mouse suprachiasmatic circadian clock in vitro. PLoS One. 2010;5(4):e10303.
19. Lee HM, Chen R, Kim H, Etchegaray JP, Weaver DR, Lee C. The period of the circadian oscillator is primarily determined by the balance between casein kinase 1 and protein phosphatase 1. Proc Natl Acad Sci U S A. 2011;108(39):16451-6.
20. Xu Y1, Padiath QS, Shapiro RE, Jones CR, Wu SC, Saigoh N, Saigoh K, Ptácek LJ, Fu YH. Functional consequences of a CKIdelta mutation causing familial advanced sleep phase syndrome. Nature. 2005;434(7033):640-4.
21. Preitner N, Damiola F, Lopez-Molina L, Zakany J, Duboule D, Albrecht U, Schibler U. The orphan nuclear receptor REV-ERBalpha controls circadian transcription within the positive limb of the mammalian circadian oscillator. Cell. 2002;110(2):251-60.
22. Sato, T.K., Panda, S., Miraglia, L.J., Reyes, T.M., Rudic, R.D., McNamara, P., Naik, K.A., FitzGerald, G.A., Kay, S.A. and Hogenesch, J.B. A functional genomics strategy reveals Rorα as a component of the mammalian circadian clock. Neuron, 2004; 43(4): 527–537.
23. André E, Conquet F, Steinmayr M, Stratton SC, Porciatti V, Becker-André M. Disruption of retinoid-related orphan receptor beta changes circadian behavior, causes retinal degeneration and leads to vacillans phenotype in mice. EMBO J. 1998;17(14):3867-77.
