# Supplementary material for: Computational modeling of the cell-autonomous mammalian circadian oscillator
Source: BMC Syst Biol. 2017 Feb 24;11(Suppl 1):27–42. doi: 10.1186/s12918-016-0379-8 (PMC5333193; doi:10.1186/s12918-016-0379-8)
Supplement: Supplementary file 2 — The comparative characteristics of mathematical models of the mammals CACO. (DOCX 22 kb) [file 12918_2016_379_MOESM2_ESM.docx]

**Additional file 2: Table S2. The comparative characteristics of mathematical models of the CO mammals.**

| Author / Ref. | Mathematical formulation | The number of variables / parameters | Genes in the model | Data & Model assumptions | Findings |
| --- | --- | --- | --- | --- | --- |
| The purpose of research: the dynamics of circadian genes | | | | | |
| Leloup and Goldbeter, 2003,  2004, 2008. 2011 | ODE | 16/53  19/70  20/73 | Bmal1, Clock, Per, Cry, Rev-erbα | The mammalian model does not distinguish among the products of the three Per genes or the two Cry genes, and instead uses single Per and Cry entities (RNA and proteins), and with phosphorylation as a mechanism controlling protein degradation.  Enzyme-substrate interactions are modeled with Michaelis-Menten type expressions, and transcription regulation is modeled by Hill-type expressions.  Light input to the model is achieved through elevation of Per levels.  Because most parameter values remain to be determined experimentally, parameter values were a semi-arbitrary chosen in a physiological range so as to yield a period of oscillations in continuous darkness (DD) close to 24 h. | Autonomous oscillations with adverse phase of Per and Bmal1 mRNAs in dark period.  The phase of circadian oscillations during entrainment in LD critically depends on the parameters that govern the level of CRY protein.  It is shown that sustained oscillations may occur even in the absence of PER protein, which proves the existence of the second oscillatory mechanism.  This model was used to address disorders of the sleep–wake cycle in humans, linked to perturbations of the circadian clock, such as the shift of phase observed for some parameter values upon entrainment by LD cycles, or the lack of entrainment in LD cycles.  A increase the maximum rate of PER phosphorylation by the protein kinase CK1ε may correspond to a delayed phase of the sleep/wake cycle in LD, and to an increase in the autonomous period of circadian oscillations in DD whereas a decreasing of the PER phosphorylation leads to the advance of the phase in LD then accompanies a decrease in autonomous period. |
| Forger and Peskin, 2003  Forger & Peskin, 2004 | ODE | 73/36 | Per1/2, Cry1/2, Rev-erb α, CkI | Fitting the model and comparison with experimental data was done using optimization procedure called a coordinate search method and data on the relative concentrations of the different clock mRNA and proteins. This model achieves a good agreement with experimental data. | Incorporating this level of detail allows to test the differential roles of mPER1 and mPER2 in phase resetting, simulate mutations in individual proteins(e.g., mPER1 or mPER2), and study specific aspects of phosphorylation (e.g., the tau mutation) or transcription regulation. The low intensity light stimuli were able to cause type 1 phase shifts where the phase response curve is continuous. High intensity light stimuli were able to cause type 0 (strong) phase shifts. Computational experiments predict accurately the set of phases in which light stimuli were ineffective (known as the dead zone). |
| Becker-Weimann et al., 2004a  Becker-Weimann et al., 2004b | ODE | 7/24  8/24 | Bmal1, Per, Cry, Rev-erb α | This mathematical model developed to reflect the essential features of the mammalian circadian oscillator to characterize the differential roles of negative (via REV-ERB) and positive feedback loops. For this purpose it was used a small system based on linear kinetics and with a high Hill coefficient, as this keeps the number of parameters in the system low. Criteria for parameter estimations in the order of their importance were: i) the existence of oscillations with a period close to 24 h; ii) correct phases between various oscillator components; and iii) reasonable peak/trough ratios of the mRNA and protein concentrations. The model also accounts for the differential effect of the Cry1^-/-^ and Cry2^-/-^ mutations on the circadian period. A reliable circadian oscillator should be robust against small variations in the reaction rates in order to function properly. | Retaining of clock gene oscillatory behavior even when the positive feedback is replaced by a constant term (Rev-Erbα mutant).  The negative feedback guarantees undisturbed circadian oscillations, whereas the easily achieved modulation of the components of the positive feedback provides the possibility to change the phase and level of clock-dependent gene transcription.  The simulations of the extended model indicate that the original model is robust with respect to the incorporation of the additional component. Depending on the kinetics of the Per2/Cry transcriptional activation by BMAL1, an increasing BMAL1 expression leads to either an increase or decrease of the clock period. |
| Forger & Peskin, 2005 | SDE | 73/36 | Per1/2, Cry1/2, Rev-erb α, CkI | This stochastic model is a direct generalization of the deterministic mammalian circadian clock model (Forger and Peskin, 2003).  Stochastic simulations used the Gillespie method, but in an efficient way.  The volume of a cell was chosen so that there are 5,000 molecules of CRY1 at its rhythm peak. | The simulations showed decreased period-to-period variability as more molecules and promoter events were present, with a 1/n^0.5^ scaling, where *n* is the number of molecules of any species.  The stochastic model can oscillate even when the corresponding deterministic model does not.  The PER2 mutant (i.e., PER2 removed) was rhythmic in this stochastic model even though the deterministic PER2 mutant was not rhythmic.  The numerical experiments shown that certain aspects of the mammalian clock’s structure might play a key role in noise immunity. Multiple copies of genes and rapid interactions with promoters are features that reduce the variability of the period of the clock. The robustness of circadian clocks appears to increase as more molecules are present or more frequent promoter interactions occur.  The numerical experiments shown that gene duplication increases robustness by providing more promoters with which the transcription factors of the model can interact. |
| Misrky et al., 2009 | ODE | 21/132 | Per1/2, Cry1/2, Rev-erb α, Clock, Bmal1, Rorc | Michaelis–Menten kinetics and Hill-function was assumed for transcriptional rates and mass action kinetics and for all other rates (e.g., mRNA and protein degradations, translation, complex formation and dissociation, etc.). The model was evaluated against the experimentally observed cell-autonomous circadian phenotypes of gene knockouts, particularly retention of rhythmicity and changes in expression level of molecular clock components. The model addresses the overlapping but differential functions of CRY1 and CRY2 in the clock mechanism: they antagonistically regulate period length and differentially control rhythm persistence and amplitude. | Predicts phenotypes of 7 single knockouts and 2 double knockout mutations of clock genes.  The model can explain kinetically why removal of the Per1, Per2, or Cry1 genes leads to arrhythmicity, whereas removal of the Cry2 gene does not have this effect. |
| Relogio *et al., 2011* | ODE | 19/71  76?? | Per (Per1/2/3)  Cry (Cry1/2)  Ror (Ror α/β/c)  Rev-Erb (Rev-Erb α/β)  Bmal (Bmal1/2) | The system of equations was assembled using mostly the law of mass action and linear degradation kinetics. Nonlinearities were introduced to describe transcription reactions by means of Michaelis-Menten kinetics and Hill functions. Many parameters could be retrieved from the literature and others were estimated based on known phases and amplitudes using linear-time-invariant systems theory. The remaining parameters were found by fitting the expression profiles of the variables to published phase and amplitude values. | Dependence of clock gene periodicity on Per mRNA degradation rate.  The model predicted that overexpression of members of the RBR loop would lead to damped or even to the loss of oscillations. These predictions was verified experimentally. |
| Kim and Forger,  2012 | ODE | 181/75 | Per1/2, Cry1/2, Rev-Erbα,  Rev-Erbβ, CKIε/δ, GSK3 β,BMAL1/2, CLOCK/NPAS2 | The parameters of the model are estimated using a simulated annealing method (a global stochastic parameter searcher) for model fitting to experimental data for WT and knockout mutation phenotypes.  The detailed model is available in Mathematica, Matlab and XPPAUT format. | The model accurately predicts the phenotype of known mutations of genes in the central circadian clock.  The stoichiometry (the average ratio between the concentrations of repressors to that of activators over a period) plays a key role in determining which mutations showed rhythmic phenotypes. The model predicted that stoichiometry provides a unifying principle to determine the rhythmicity of mutations of the mammalian circadian clock. An approximate range of the stoichiometries that permit oscillations was derived.  The two different designs for robust biological oscillators was described: (1) the negative feedback loop is suitable for biological clocks in which the maintenance of a fixed period is crucial (e.g., circadian clocks); (2) the positive feedback loop is suitable for the biological oscillators that need to tune their period (e.g., cell cycle or pacemaker in the sinoatrial node).  It was predicted that tight binding between activators and repressors is required for rhythmicity. Point mutations in binding sites can generate different binding affinities between PER–CRY and BMAL1–CLOCK. Comparing the experimentally measured binding affinities of these mutants, with the resultant rhythms, or lack thereof, would directly test this prediction. |
| Jolley Craig C, et al, 2014 | ODE | 10/21 | Dbp/Tef/Hlf  Ror α/β  Cry1  Rev-Erb α/β  E4bp4 | Transcriptional regulation involves three clock-controlled elements: the E/E’-box, which governs morning expression; the D-box, which promotes daytime expression; and the RRE, which leads to evening expression.  The model more correctly describes the transcriptional regulation of Cry1 by the D-box and RRE (Rev-Erbα/ROR response element) promoter elements and allows for ensemble-based predictions of phase response curves. The model have been validated using differential evolution for optimization. | The model reproduces predictions concerning the dual regulation of Cry1 by the D-box and Rev-ErbA/ROR response element (RRE) promoter elements and allows for ensemble-based predictions of phase response curves (PRCs). Nonphotic signals such as Neuropeptide Y (NPY) may act by promoting Cry1 expression, whereas photic signals likely act by stimulating expression from the E/E' box. Ensemble generation with parameter probability restraints reveals more about a model's behavior than a single optimal parameter set. |
| Korenčič et al., 2012, 2014 | DDE | 6/50 | Bmal1, Rev-erbα, Per2, Cry1, Rorg, and Dbp | The model is based on own quantitative time resolved RT-PCR data in mouse liver in constant darkness and under light-dark cycles. The model is a direct translation of experimental observations to DDEs composed of production and degradation terms and includes just three clock-controlled elements (E-box, D-box, and RRE) and no systemic regulation. Despite this simplicity, the model reproduces the experimentally measured phases, amplitudes, and waveforms. | The amplitude and phase of cytochromes p450 can be reproduced by our models with variations in the core clock.  The multiplicative effect of E-boxes, D-boxes and RRE leads to appearance of harmonics that are different from 24 hours. |
| Yan et al., 2014 | ODE | 20/62 | *Bmal1, Per1, Cry1, Per2, Cry2*, *Rev-erba*) | The model was applied to study how a negative primary feedback loop is associated with some ‘auxiliary’ loops and how these interlocking loops coordinate regulate the period and maintain its robustness;  In order to focus on the transcriptional regulations, they assumed that the post-translational time delay of each gene is fixed as an explicit time delay.  The model includes the following process:  (1) The regulations of PLBS activity and RORE activity  (2) Transcriptions of Per1, Per2, Cry1, Cry2, Bmal1, Rev-erba genes  (3) Translation of Per1 mRNA, Per2 mRNA, Cry1 mRNA, Cry2 mRNA, Bmal1 mRNA, Rev-erba mRNA  (4) The post-translational regulations.  The comparison of period length between the experimental data and the simulation result was carry out on follow genotype: WT, *Per1^-/-^*, *Per2^-/-^, Cry1^-/-^, Cry2^-/-^, Rev-erbα^-/-^ , Fbxl3^-/-^, Rev-erbα^-/-^/Fbxl3^-/-^, Bmal1^-/-^ .* | The post-translational time delays are the main factors, which significantly change the period of the oscillation. The intensity ratio of the primary loop to the auxiliary loop is inversely related to the period length, even when post-translational feedback is fixed. |

1. Leloup JC1, Goldbeter A. Toward a detailed computational model for the mammalian circadian clock. Proc Natl Acad Sci U S A. 2003;100(12):7051-6.
2. Leloup JC1, Goldbeter A Modeling the mammalian circadian clock: sensitivity analysis and multiplicity of oscillatory mechanisms. J Theor Biol. 2004;230(4):541-62.
3. Leloup JC, Goldbeter A. Modeling the circadian clock: from molecular mechanism to physiological disorders. Bioessays. 2008;30(6):590-600
4. Leloup JC1, Goldbeter A. Critical phase shifts slow down circadian clock recovery: implications for jet lag. J Theor Biol. 2013;333:47-57.
5. Forger DB, Peskin CS. A detailed predictive model of the mammalian circadian clock. Proc Natl Acad Sci U S A. 2003;100(25):14806-11.
6. Forger DB, Peskin CS. Model based conjectures on mammalian clock controversies. J Theor Biol. 2004;230(4):533-9.
7. Becker-Weimann S, Wolf J, Kramer A, Herzel H. A model of the mammalian circadian oscillator including the REV-ERBalpha module. Genome Inform. 2004a;15(1):3-12.
8. Becker-Weimann S, Wolf J, Herzel H, Kramer A. Modeling feedback loops of the Mammalian circadian oscillator. Biophys J. 2004b Nov;87(5):3023-34.
9. Forger DB, Peskin CS Stochastic simulation of the mammalian circadian clock.Proc Natl Acad Sci U S A. 2005;102(2):321-4.
10. Mirsky HP, Liu AC, Welsh DK, Kay SA, Doyle FJ 3rd. A model of the cell-autonomous mammalian circadian clock. Proc Natl Acad Sci U S A. 2009; 106:11107–11112. [PubMed:19549830]
11. Relogio A, Westermark PO, Wallach T, Schellenberg K, Kramer A, Herzel H. Tuning the mammalian circadian clock: robust synergy of two loops. PLoS Comput Biol. 2011; 7:e1002309. [PubMed: 22194677]
12. Kim JK, Forger DB A mechanism for robust circadian timekeeping via stoichiometric balance. Mol Syst Biol. 2012;8:630.
13. Jolley CC , Ukai-Tadenuma U, Perrin D, Ueda HR Mammalian Circadian Clock Model Incorporating Daytime Expression Elements // Biophysical Journal. 2014. V. 107. 1462–1473
14. Yan J, Shi G, Zhang Z, Wu Xi, Liu Z, Xing L, Qu Zhipeng, Dong Z, Yang Ling and Xu Y An intensity ratio of interlocking loops determines circadian period length // Nucleic Acids Research, 2014, Vol. 42, No. 16. 10278–10287
